# Fibroblasts are the most suitable cell source for regenerative medicine due to their high intracellular fibroblast growth factor 2 content

**DOI:** 10.1016/j.bbrep.2023.101510

**Published:** 2023-07-05

**Authors:** Masashi Yanagihara, Yutaro Matsuno, Koji Ueno, Hiroshi Kurazumi, Ryo Suzuki, Toshiki Tanaka, Kimikazu Hamano

**Affiliations:** Department of Surgery and Clinical Science, Yamaguchi University Graduate School of Medicine, 1-1-1 Minami-kogushi, Ube, 755-8505, Japan

**Keywords:** Fibroblasts, Fibroblast growth factor 2, Cell sheets, Regenerative medicine, Dry-preservation

## Abstract

In our previous study, we found that dry-preserved multilayered fibroblast cell sheets promoted angiogenesis and wound healing in a mouse ulcer model by releasing high levels of intracellular fibroblast growth factor 2 (FGF2), hepatocyte growth factor (HGF), and vascular endothelial growth factor (VEGF), from dried cells. In the present study, to identify which cell types are suitable for human dry-preserved cell sheets (dry sheets), we compared the intracellular FGF2 levels in seven types of cells reported as cell sheets for clinical use or preclinical studies. FGF2 levels were high in mesenchymal cells, including human oral fibroblasts (HOFs) and human dermal fibroblasts (HDFs), human dental pulp stem cells (DPSCs), and human mesenchymal stem cells (MSCs); in contrast, FGF2 levels in human umbilical vascular endothelial cells (HUVECs), human skeletal muscle myoblasts (SkMMs), and human epidermal keratinocytes (HEKs) were remarkably low, approximately 25% those in fibroblasts. In addition, we prepared dry sheets from HOFs, DPSCs, and MSCs, and analyzed the growth factors released from each dry sheet upon rehydration. High levels of FGF2, HGF, and VEGF were detected in the eluate prepared by immersing each dry sheet. In particular, FGF2 and HGF were the most abundant in HOFs. An *in vitro* cell proliferation assay showed that these eluates significantly enhanced HUVEC proliferation compared to control cells. Furthermore, cells incubated with HOF eluate showed significantly higher cell proliferation than cells incubated with DPSC and MSC eluates. However, this proliferative response was significantly blocked by FGF2-neutralizing antibodies. These results demonstrate that growth factors released from human dry sheets have physiological activity and that this activity is mainly mediated by the effect of FGF2. Fibroblasts are ideal for the clinical application of dry-preserved cell sheets in humans owing to their high intracellular FGF2 content, fast cell proliferation, ease of handling, availability, and low culture costs, making them the most suitable cell source for regenerative medicine, with FGF2 release as the mechanism of action.

## Highlights

• Releasing FGF2 from dried cells plays a major therapeutic role in dried cell sheets.
• Fibroblasts had the highest FGF2 content among cells reported as human cell sheets.
• High FGF2 levels were released from dried fibroblast cell sheets upon rehydration.
• FGF2 released from dried fibroblast cell sheets promoted HUVEC proliferation.
• Fibroblasts are ideal cells for clinical applications of dry-preserved cell sheets.

## 1 Introduction

Cell sheet transplantation is a cell-based therapy with potential applications in regenerative medicine [1]. We have previously reported that multilayered fibroblast cell sheets promote angiogenesis and wound healing, mediated by the paracrine effect of the growth factors they secrete, and are effective in the treatment of cutaneous ulcers and the prevention of postoperative bronchopleural and pancreatic fistulas in animal models [[2], [3], [4], [5]].

For the industrialization and widespread use of cell sheet transplantation therapy, it is essential to reduce manufacturing costs through off-the-shelf mass production using allogeneic cells and develop a method of cell sheet preservation that enables convenient use. In cell-based therapies, cryopreservation is considered an optimal method for the long-term storage and transport of viable cells [6]. Therefore, cryopreservation methods for cell sheets have been investigated [[7], [8], [9], [10], [11]]. However, there are still problems in terms of cost and facilities, including cold-chain storage and transportation, due to cell damage during freezing and thawing and the need for special equipment during freezing [9,10].

Previously, we focused on dry storage as a more convenient method for cell sheet preservation and conducted a basic study on dry-preserved cell sheets prepared by air-drying multilayered fibroblast cell sheets. Although the cells composing the dry-preserved cell sheets were dead cells whose cell membranes were damaged by drying, the dry-preserved cell sheets promoted angiogenesis and wound healing in a diabetic mouse model of full-thickness skin defect [12]. Upon rehydration, dry-preserved cell sheets release high levels of fibroblast growth factor 2 (FGF2/basic FGF), vascular endothelial growth factor (VEGF), and hepatocyte growth factor (HGF). These released growth factors exhibited physiological activity, primarily due to the action of FGF2, as shown by *in vitro* inhibition experiments using FGF2-neutralizing antibodies [12]. It is well known that FGF2 is a potent growth factor and is important for promoting wound healing and angiogenesis [13]. However, there have been no reports on FGF2 as an effector secreted from living cell sheets because its extracellular secretion is generally inefficient owing to the lack of secretory signal peptides. Previous studies have demonstrated that the major release of FGF2 is caused by damaged or dead cells [[14], [15], [16], [17]].

While conventional living cell sheets have been focused on and characterized by sustained secretion of growth factors from transplanted cells [[2], [3], [4], [5],^18^,^19^], in dry-preserved cell sheets, intracellular FGF2 plays an important role in mediating therapeutic effects [12]. However, only a few studies have compared intracellular FGF2 levels in different cell types used as cell sheets, and the cell types suitable for dry-preserved cell sheets remain unknown. In this study, we compared the intracellular FGF2 levels in cell types that have been studied as cell sheets to determine which cell types are suitable for human dry-preserved cell sheets.

## 2 Materials and methods

### 2.1 Cells

Human oral fibroblasts (HOFs) were isolated from the oral tissues of healthy volunteers (n = 3). This study was approved by the Institutional Review Board Committee of Yamaguchi University Hospital (#H26-111) and conducted in accordance with the Declaration of Helsinki. Informed consent was obtained from all study participants prior to their participation in the study.

HOFs and human dermal fibroblasts (HDFs; KF-49009; Kurabo, Osaka, Japan) were cultured in CTS™ AIM-V™ medium (Thermo Fisher Scientific, Waltham, MA, USA) supplemented with 10% fetal bovine serum (FBS; Thermo Fisher Scientific). HOFs and HDFs were seeded at a density of 2500 cells/cm^2^ and cultured for three days. HOFs and HDFs at passage five were harvested and used for the experiments. Human mesenchymal stem cells derived from the bone marrow (MSCs; PT-2501; Lonza, Basel, Switzerland) were cultured using MSCBM™ Basal Medium (PT-3238; Lonza) supplemented with 10% mesenchymal cell growth supplement (MCGS), 2% l-glutamine, and 0.1% Gentamicin/Amphotericin-B (MSCGM SingleQuots™ Kit, PT-4105; Lonza). MSCs were seeded at a density of 5000 cells/cm^2^. The medium was changed after 24 h of initiating culture and every three days thereafter. MSCs were passaged when they reached 90% confluency. MSCs at passage four were harvested and used in the experiments. Human dental pulp stem cells (DPSCs; PT-5025l; Lonza) were cultured using DPSC Basal Medium (PT-3927; Lonza) supplemented with 10% dental pulp stem cell growth supplement (DPSCGS), 2% l-glutamine, 1% ascorbic acid, and 0.1% gentamicin/amphotericin-B (DPSC SingleQuots™ Kit, PT-4516; Lonza). DPSCs were seeded at a density of 5000 cells/cm^2^. The medium was changed after 24 h of initiating culture and every three days thereafter. DPSCs were passaged when they reached 90% confluence. DPSCs at passage three were harvested and used in the experiments. Human umbilical vein endothelial cells (HUVECs; C-12206; PromoCell GmbH, Heidelberg, Germany) were cultured using Endothelial Cell Growth Medium 2 (C-22011; PromoCell) supplemented with 2% fetal calf serum, 5 ng/mL hEGF, 10 ng/mL basic FGF, 20 ng/mL IGF, 0.5 ng/mL VEGF, 1 μg/mL ascorbic acid, 22.5 μg/mL heparin, and 0.2 μg/mL hydrocortisone (ECGM). HUVECs were seeded at a density of 5000 cells/cm^2^. The medium was changed after 24 h of initiating culture and every three days thereafter. HUVECs were passaged when they reached 90% confluency. HUVECs at passage three were harvested and used for the experiments. Human skeletal muscle myoblasts (SkMMs; CC-2580; Lonza) were cultured in SkBM™-2 Basal Medium (CC-3246; Lonza) supplemented with 0.1% hEGF, 0.1% dexamethasone, 2% l-glutamine, 10% FBS, and 0.1% gentamicin/amphotericin-B (SkGM™-2 SingleQuots™ Kit, CC-3244; Lonza). SkMM cells were seeded at a density of 3500 cells/cm^2^. The medium was changed after 24 h of initiating culture and every other day thereafter. SkMM cells were passaged when they reached 70% confluency. SkMM cells at passage three were harvested and used for the experiments. Human epidermal keratinocytes (HEKs; KK-4009; Kurabo) were cultured in HuMedia-KB2 (KK–2350S; Kurabo) basal medium supplemented with 10 μg/mL insulin, 0.1 ng/mL hEGF, 0.67 μg/mL hydrocortisone hemisuccinate, 50 μg/mL gentamicin, 50 ng/mL amphotericin-B, and 0.4% V/V bovine pituitary extract (BPE) (KK-6150; Kurabo). HEK cells were seeded at a density of 2500 cells/cm^2^. The medium was changed after 24 h of initiating culture and every other day thereafter. HEK cells were passaged when they reached 70% confluency. HEK cells at passage three were harvested and used for the experiments. Details of the cells used in this study are listed in Table 1. All cells were cultured in 10-cm dishes (60 cm^2^) containing 12 mL of complete medium and maintained at 37 °C in a humidified incubator with 5% CO_2_.

**Table 1:** Details of cells used in this study.

Cell types	Lot number	Age	Sex	Race
Human oral fibroblasts (HOFs)	Isolate #1	32	Male	Japanese
	Isolate #2	34	Male	Japanese
	Isolate #3	37	Male	Japanese
Human dermal fibroblasts (HDFs)	#00978	Newborn	Male	Black/African American
Human Dental pulp stem cells (DPSCs)	20TL365542	29	Female	Black/African American
Human mesenchymal stem cells (MSCs)	20TL168582	26	Male	Hispanic
Human umbilical vein endothelial cells (HUVECs)	436Z001	Newborn	Male	Black/African American
Human skeletal muscle myoblasts (SkMMs)	20TL356515	36	Male	Caucasian
Human epidermal keratinocytes (HEKs)	#07401	Newborn	Male	Asian

### 2.2 Cell extracts

Cells (5 × 10^5^) were collected in 1.5-mL microtubes and resuspended in 200 μL of Dulbecco's modified Eagle medium (DMEM; Thermo Fisher Scientific). The cells were placed in a deep freezer (−80 °C) for 60 min, followed by thawing on ice for 90 min. The freeze-thaw cycles were repeated twice to destroy the cell membranes. The samples were centrifuged at 2460×*g* for 5 min at 4 °C, and the supernatants were collected and stored at −30 °C until assays were performed.

### 2.3 Preparation of dry-preserved cell sheets

Dry-preserved cell sheets were prepared from HOFs, DPSCs, and MSCs, as described previously [12]. Briefly, the cells were seeded in a 24-well plate (5 × 10^5^ cells/well) using 2 mL of medium consisting of CTS™ AIM-V™ and HFDM-1 (+) (Cell Science & Technology Institute, Sendai, Miyagi, Japan) supplemented with 5% FBS and incubated at 37 °C in a humidified incubator under 5% CO_2_ for three days. After incubation, the cell sheets were rinsed twice with 2 mL of phosphate-buffered saline (PBS) and then incubated with 500 μL of dispase solution (10 PU/mL; FUJIFILM Wako Pure Chemical Corporation, Osaka, Japan) for 30 min at 37 °C in a humidified incubator under 5% CO_2_. After washing twice with PBS, the cell sheets were gently detached from the culture plates. These sheets were transferred onto a silicon pedestal using a 1000-μL wide-bore tip and unfolded to avoid wrinkling. After removing as much water as possible, the sheets were left to stand for 30 min. The dry-preserved cell sheets were peeled off from the silicon with tweezers and transferred to 1.5-mL microtubes.

### 2.4 Eluates of dry-preserved cell sheets

Eluates of dry-preserved cell sheets were prepared as previously described [12]. Briefly, each dry-preserved cell sheet was immersed in 200 μL of DMEM in a 1.5-mL microtube with a sealed lid for 24 h at 37 °C in a humidified incubator with 5% CO_2_. After incubation, the samples were centrifuged at 2460×*g* for 5 min at 4 °C, and the supernatants were collected and stored at −30 °C until the assays were performed.

### 2.5 Enzyme-linked immunosorbent assay (ELISA)

The concentration of FGF2, HGF, and VEGF in the samples was measured using human Quantikine ELISA Kits (DFB50 for FGF2, DHG00B for HGF, DVE00 for VEGF; R&D Systems, Inc. Minneapolis, MN, USA) according to the manufacturer's instructions. All samples were assayed in triplicate. The total protein concentration in each sample was determined by the Coomassie (Bradford) Protein Assay Kit (23200; Thermo Fisher Scientific) with bovine serum albumin as standard.

### 2.6 Cell proliferation assay

HUVECs were seeded in 96-well plates at 2000 cells/well in 100 μL Endothelial Cell Growth Medium 2 with supplements (ECGM), followed by the addition of 100 μL of either the eluate sample diluted by 5 folds with DMEM or DMEM without FBS. After 48 h of incubation at 37 °C in a humidified incubator with 5% CO_2_, the culture supernatant was removed followed by the addition of 100 μL of ECGM containing 10% [2-(2-methoxy-4-nitrophenyl)-3-(4-nitrophenyl)-5-(2,4-disulfophenyl)-2H-tetrazolium, monosodium salt] (WST-8) reagent (Cell Count Reagent SF; Nacalai Tesque, Inc. Kyoto, Japan). After 2 h of incubation, the absorbance of the supernatant was measured at 450 nm (reference wavelength: 630 nm). The cell proliferation rate was calculated using HUVECs cultured in DMEM as the control.

### 2.7 Neutralizing antibody experiment

HOFs dry sheet eluate sample was diluted 5 folds with DMEM. DMEM containing recombinant FGF2 protein (rFGF2, 0 or 10 ng/mL, 062–06661; FUJIFILM Wako) was also prepared. These samples were incubated for 60 min at 37 °C with the anti-FGF-2 neutralizing antibody (30.3 μg/mL, #05–117, clone bFM-1; Merck Millipore, Darmstadt, Germany), or the control antibody (Mouse IgG1 isotype control, clone11711; 30.3 μg/mL, MBA002; R&D Systems). HUVECs were seeded in 96-well plates at 2000 cells per well in 100 μL ECGM, followed by the addition of 100 μL of either the eluate sample with the antibodies or DMEM containing rFGF2 (0 or 10 ng/mL) with antibodies. After 48 h of incubation at 37 °C in a humidified incubator with 5% CO_2_, the culture supernatant was removed followed by the addition of 100 μL of ECGM containing the 10% WST-8 reagent. After 2 h of incubation, the absorbance of the supernatant was measured at 450 nm (reference wavelength: 630 nm). The cell proliferation rate was calculated using HUVECs cultured in 0 ng/mL rFGF2 without antibody as the control.

### 2.8 Statistical analysis

All data are presented as mean ± standard deviation. Statistical differences were assessed by one-way analysis of variance followed by the Tukey–Kramer test for multiple comparisons between groups. Data were statistically analyzed in StatFlex (ver6; Artech Inc., Osaka, Japan). Statistical significance was set at P < 0.05.

## 3 Results

### 3.1 Mesenchymal cells, including fibroblasts, store high intracellular levels of FGF2

**Fig. 1:**
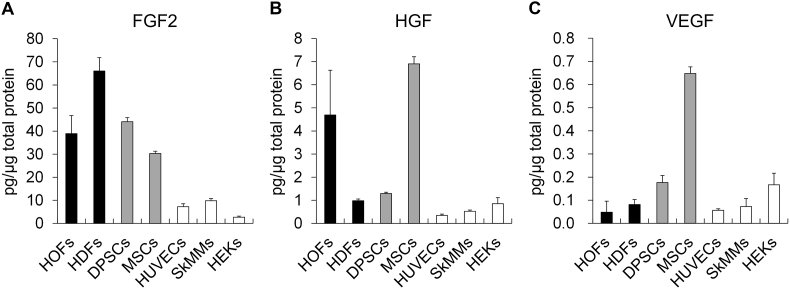
Comparison of growth factors in freeze-thaw extracts of various cells used as cell sheets. FGF2 (A), HGF (B), and HGF (C) levels in cell extracts obtained by freeze-thawing were measured by ELISA and corrected for total protein content. HOFs, human oral fibroblasts; HDFs, human dermal fibroblasts; DPSCs, human dental pulp stem cells; MSCs, human mesenchymal stem cells; HUVECs, human umbilical vein endothelial cells; SkMMs, human skeletal muscle myoblasts; HEKs, human epidermal keratinocytes.

The cell contents were extracted by freeze-thawing, and the amount of FGF2 per μg of total protein was compared. FGF2 levels were high in mesenchymal cells, including fibroblasts and mesenchymal stem cells such as DPSCs and MSCs, and low in HUVECs, SkMMs, and HEKs, which were approximately 1/4 or lower compared to those of fibroblasts (Fig. 1A). The amounts of HGF and VEGF in these cell extracts were also analyzed. HGF content was the highest in MSCs, followed by HOFs, and low in other cell extracts (Fig. 1B). VEGF content was high in MSCs but low in the other cell extracts (Fig. 1C). In terms of the amount per ug of protein of the three growth factors assessed in mesenchymal cells, FGF2 was present at a level of approximately 40 pg/μg, whereas HGF was present at a level of 4 pg/μg, approximately 1/10 that of FGF2, and VEGF was present at a lower level of approximately 1 pg/μg or lower.

### 3.2 The amount of FGF2 released from dry-preserved cell sheets

**Fig. 2:**
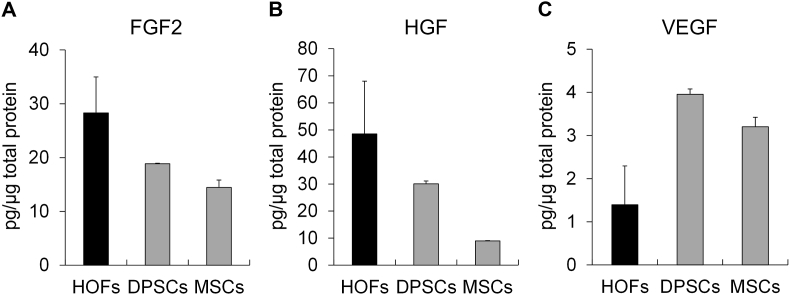
Comparison of growth factors released from dry sheets. FGF2 (A), HGF (B), and HGF (C) levels in the supernatant of dry sheets immersed in solution were measured by ELISA and corrected for total protein content. HOFs, human oral fibroblasts; DPSCs, human dental pulp stem cells; MSCs, human mesenchymal stem cells.

Dry-preserved cell sheets were prepared from HOFs, DPSCs, and MSCs, and the amount of FGF2, VEGF, and HGF per μg of total protein released into the solution was determined. HOFs had the highest levels of FGF2, which were 72.8% of those in cell extracts obtained by freeze-thawing, followed by DPSCs and MSCs, which were 42.9% and 47.9% of those in cells extracts obtained by freeze-thawing, respectively (Fig. 2A). Moreover, HOFs and DPSCs showed a high HGF content of 48.5 pg/μg and 30.1 pg/μg, which was 10.3- and 23.3-fold higher than that in the cell extracts, respectively (Fig. 2B), and MSCs showed an HGF content of 8.95 pg/μg, which was 1.30-fold higher than that in the cell extracts. VEGF content was the highest in DPSCs, followed by MSCs and HOFs, which was higher than that in the cell extracts (Fig. 2C).

### 3.3 Dry-preserved cell sheet eluate promotes cell proliferation in HUVECs

**Fig. 3:**
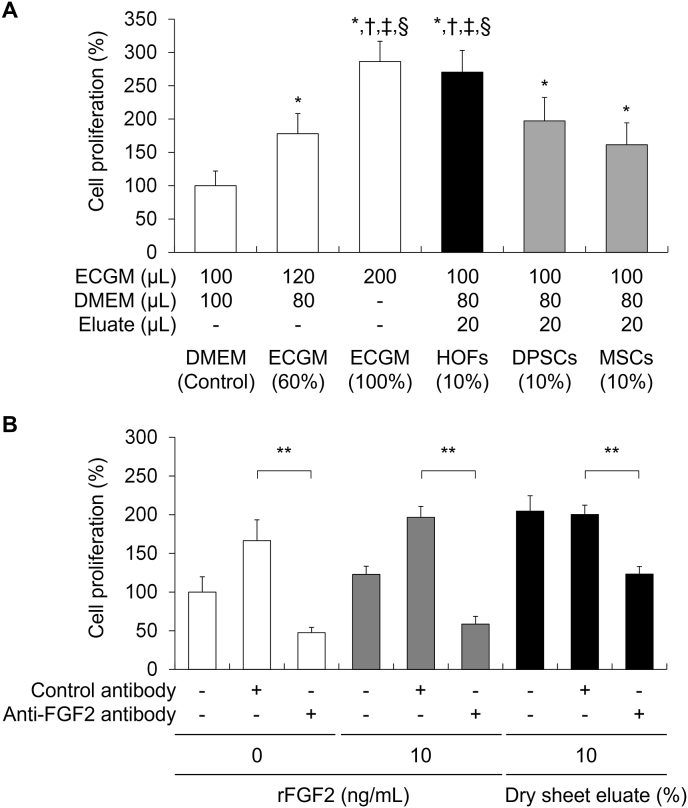
Proliferation of human umbilical vein endothelial cells (HUVECs) in response to incubation with the dry sheet eluate and the effect of FGF2. (A) Proliferative response of HUVECs to dry sheet eluates prepared from HOFs, DPSCs, and MSCs. After 48 h incubation with 10% dry sheet eluates, cell proliferation was analyzed using the WST-8 reagent. The cell proliferation rate was calculated using HUVECs cultured in DMEM as the control. N = 9 per group, *P < 0.01 versus DEME (Control), †P < 0.01 versus ECGM (60%), ‡P < 0.01 versus DPSCs, §P < 0.01 versus MSCs. ECGM, Endothelial Cell Growth Medium 2 with supplements; HOFs, human oral fibroblasts; DPSCs, human dental pulp stem cells; MSCs, human mesenchymal stem cells; HUVECs, human umbilical vein endothelial cells. (B) Effect of FGF2 neutralization on the proliferative response of HUVECs induced by 10% HOFs dry sheet eluates. After 48 h of incubation with FGF2-neutralized HOFs dry sheet eluates, cell proliferation was assessed using the WST-8 reagent. The cell proliferation rate was calculated using HUVECs cultured in 0 ng/mL rFGF-2 without antibody as the control. n = 9 per group, **P < 0.01. rFGF-2, recombinant FGF-2 protein.

To investigate the bioactivity of the dry sheet eluate, we assessed the cell proliferation of HUVECs *in vitro*. HUVECs were cultured for 48 h with a medium and the 5-fold diluted eluate sample mixed at a 1:1 ratio. As shown in Fig. 3A, the proliferation of cells incubated with the dry sheet eluate of HOFs, DPSCs, and MSCs was 2.70, 1.97, and 1.61 times higher than that of the control cells, respectively. In addition, the proliferation of cells incubated with the HOF dry sheet eluate was significantly higher than that of cells incubated with the dry sheet eluates of DPSCs and MSCs. These results show that the dry sheet eluates exhibited biological effects on cells.

### 3.4 FGF2-neutralizing antibodies present in the dry sheet eluate prevent cell proliferation of HUVECs

FGF2 is a potent growth factor, and the dry sheet eluate contained a large amount of FGF2, suggesting that FGF2 may attribute to the biological activity of the dry sheet eluate. Therefore, we investigated whether FGF2 in the eluate directly affected HUVECs proliferation using a neutralizing antibody against FGF2 or recombinant FGF2 protein (rFGF2). The proliferation of HUVECs was promoted in the HOF dry sheet eluate or rFGF2 groups treated with control antibodies. In contrast, these proliferative responses were significantly inhibited by FGF2 neutralization (Fig. 3B). These results demonstrate that the biological activity of the dry sheets was mainly mediated by FGF2.

## 4 Discussion

In a previous study, we focused on dry storage as a new method of cell sheet preservation and investigated a dried cell sheet obtained by air-drying multilayered mouse fibroblast cell sheets [12]. The dry-preserved cell sheets showed significant wound closure in a whole skin defect model of diabetic mice compared to the untreated group. We investigated the mechanism of action and found that FGF2, a potent cell growth factor not secreted by living cells, was released at high levels from the dry-preserved cell sheets and acted on peri-wound cells. In the present study, we compared various cell types, using the intracellular FGF2 level as an indicator to identify optimal cells for the clinical application of dry-preserved cell sheets. FGF2 was the most abundant in mesenchymal cells, including HOFs, HDFs, DPSCs, and MSCs. In contrast, intracellular FGF2 levels in HUVECs, SkMMs, and HEKs were remarkably low compared with those in fibroblasts. Consistent with the results of our study, Aizman et al. reported that the amount of intracellular FGF2 is high in the order of HDF > MSC > HUVEC [17]. According to the Human Protein Atlas database [20] (Version 21.1, https://www.proteinatlas.org/ENSG00000138685-FGF2, [accessed November 17, 2022]), single-cell RNA-seq analysis of various human tissues revealed that fibroblasts had the second-highest expression of FGF2 mRNA, following that in astrocytes. RNA-seq of human cell lines found in the Human Protein Atlas also showed high FGF2 mRNA expression in fibroblasts derived from the foreskin (BJ, 243.8 normalized transcripts per million [nTPM]; BJ hTERT+, 148.4 nTPM; fHDF/TERT166, 278.2 nTPM) but low FGF2 mRNA expression in vascular endothelial cells (HUVEC TERT2, 11.8 nTPM; TIME; 10.6 nTPM), skeletal muscle cells (HSkMC [primary], 16.8 nTPM) and the keratinocyte cell line HaCaT (0.7 nTPM). Furthermore, the amount of FGF2 released from the dry-preserved cell sheets was approximately 42.9–72.8% of that of the intracellular content. Conversely, it is suggested that if cells with low FGF2 content are used as dry-preserved cell sheets, sufficient FGF2 release cannot be expected. In terms of clinical application, ideal cells for dry-preserved cell sheets should have high intracellular FGF2 content, as well as rapid cell proliferation, easy handling, availability, and low culture costs. Therefore, fibroblasts are the most suitable cells for the clinical application of dry-preserved cell sheets.

In this study, dry-preserved cell sheets prepared with HOFs, DPSCs, and MSCs released FGF2, HGF, and VEGF upon rehydration, as in previous mouse experiments [12]. In particular, FGF2 and HGF were the most abundant in HOFs. HGF and VEGF were detected in the eluate of dry-preserved cell sheets at levels approximately 10-fold higher than those in cell extracts. The HGF and VEGF production capacity of fibroblasts is enhanced by culturing the cells as multilayered cell sheets rather than culturing them in a monolayer [2,3]. In contrast to previous studies comparing the secretory capacity in the culture supernatant, the present study revealed differences even at the intracellular protein level. Furthermore, these eluates significantly promoted the proliferation of HUVECs compared to the control group, and this proliferative response was suppressed by the FGF2-neutralizing antibody. These results indicate that, in human cells, growth factors released from dry-preserved cell sheets have physiological activity and that this activity is mainly an effect of FGF2. In our previous study in mice, the dry-preserved cell sheet eluate stimulated the secretion of HGF and VEGF from fibroblasts, in addition to promoting cell proliferation of fibroblasts. In a diabetic mouse model of whole skin defects, the allogeneic dry-preserved cell sheet treatment group exhibited significantly enhanced wound healing compared to the untreated group and showed no significant difference compared to the allogeneic living cell sheet treatment group. Histological analysis also revealed a significant increase in epidermal cell layers and areas of vascular endothelial cells at the wound edges on day 3 in the allogeneic dry-preserved cell sheet treatment group [12]. These previous and present findings reveal that human dry-preserved cell sheets may similarly promote wound healing by acting on peri-wound cells.

A limitation of this study is that the diversity of cell types used was low, with three isolates for HOFs and one each for the others. However, the results obtained in this study were consistent with previous reports and mRNA expression analysis based on databases, suggesting that increasing the number of cell types examined would not alter the fact that fibroblasts contain high intracellular levels of FGF2.

In conclusion, fibroblasts are ideal for the clinical application of dry-preserved cell sheets to humans owing to their high intracellular FGF2 content, fast cell proliferation, ease of handling, availability, and low culture costs. Therefore, fibroblasts are the most suitable cell source for regenerative medicine, with FGF2 release as the mechanism of action.

## Funding

This work was supported by a JSPS Grant-in-Aid for Scientific Research (C) (21K08845 to M.Y. and 22K08960 to R.S.), TERUMO LIFE SCIENCE FOUNDATION (to M.Y.), and The Kurata Grants by The 10.13039/501100012009Hitachi Global Foundation (to M.Y.).

## Declaration of competing interest

The authors declare that they have no known competing financial interests or personal relationships that could have appeared to influence the work reported in this paper.

## Data Availability

Data will be made available on request.
